# Glucocorticoid discontinuation rate and risk factors for relapses in a contemporary cohort of patients with giant cell arteritis

**DOI:** 10.1007/s00296-023-05527-8

**Published:** 2024-02-01

**Authors:** Christina Tsalapaki, Argyro Lazarini, Evaggelia Argyriou, Vassiliki Dania, Kyriaki Boki, Gerasimos Evangelatos, Alexios Iliopoulos, Maria Pappa, Petros P. Sfikakis, Maria G. Tektonidou, Athanasios Georgountzos, Euripidis Kaltsonoudis, Paraskevi Voulgari, Alexandros A. Drosos, Evaggelos Theotikos, Charalampos Papagoras, Theodoros Dimitroulas, Alexandros Garyfallos, Evaggelia Kataxaki, Georgios Vosvotekas, Dimitrios Boumpas, Emilia Hadziyannis, Dimitrios Vassilopoulos

**Affiliations:** 1 School of Medicine, General Hospital of Athens “Hippokration”, 2nd Department of Medicine and Laboratory, Clinical Immunology—Rheumatology Unit, National and Kapodistrian University of Athens, 114 Vass. Sophias Ave., 115 27 Athens, Greece; 2 General Hospital “Asklepieio”, Rheumatology Clinic, Athens, Greece; 3 General Hospital “Sismanogleio”, Rheumatology Clinic, Athens, Greece; 4 General Hospital NIMTS, Rheumatology Clinic, Athens, Greece; 5 School of Medicine, General Hospital “Laiko”, 1st Department of Propedeutic Internal Medicine, National and Kapodistrian University of Athens, Athens, Greece; 6 General Hospital “Gennimatas”, Rheumatology Clinic, Athens, Greece; 7 University of Ioannina, Rheumatology Clinic, Ioannina, Greece; 8 First Department of Internal Medicine, University Hospital of Alexandroupolis, Democritus University of Thrace, Alexandroupolis, Greece; 9 4th Department of Medicine, Aristotle University, Thessaloniki, Greece; 10 Thriasio General Hospital, Rheumatology Clinic, Elefsina, Greece; 11 Thessaloniki, Greece; 12 School of Medicine, 4th Department of Medicine, National and Kapodistrian University of Athens, Attikon University Hospital, Athens, Greece

**Keywords:** Giant cell arteritis, Relapse, Glucocorticoid, Vasculitis

## Abstract

The rates of relapses and therapy discontinuation in patients with giant cell arteritis (GCA) in the modern therapeutic era have not been defined. We aimed to evaluate the glucocorticoid (GC) discontinuation rate and the factors associated with relapses in a contemporary GCA cohort. Patient and treatment data were collected cross-sectionally at first evaluation and 2 years later (second evaluation), in a multicenter, prospective GCA cohort. Predictors of relapses were identified by logistic regression analyses. 243 patients with GCA were initially included (67% women, mean age at diagnosis: 72.1 years, median disease duration: 2 years) while 2 years later complete data for 160 patients were available and analyzed. All patients had received GCs at diagnosis (mean daily prednisolone dose: 40 mg) while during follow-up, 37% received non-biologic and 16% biologic agents, respectively. At second evaluation, 72% of patients were still on therapy (GCs: 58% and/or GC-sparing agents: 29%). Relapses occurred in 27% of patients during follow-up; by multivariable logistic regression analysis, large vessel involvement at diagnosis [odds ratio (OR) = 4.22], a cardiovascular event during follow-up (OR = 4.60) and a higher initial GC daily dose (OR = 1.04), were associated with these relapses. In this large, real-life, contemporary GCA cohort, the rates of GC discontinuation and relapses were 40% and 27%, respectively. Large vessel involvement, a higher GC dose at diagnosis and new cardiovascular events during follow-up were associated with relapses.

## Introduction

Giant cell arteritis (GCA) is the most common systemic vasculitis in adults affecting large arteries, especially the aorta and its branches [1]. It is a heterogenous disease, often not conforming to a single clinical presentation [2]. Ischemic complications of the arteries supplying the optic nerves result in vision loss in 15–20% of cases requiring urgent treatment [3, 4]. Glucocorticoids (GCs) remain the mainstay of treatment for GCA and should be initiated promptly to all patients [5]. While symptoms respond to therapy, relapses are common and long-term therapy with GCs is often needed, resulting in significant morbidity and mortality [6].

Large epidemiological and retrospective studies have shown that 40–79% of patients will relapse at least once during the disease course, while 40% will not be able to discontinue GCs due to multiple relapses [6, 7]. Relapses are more frequent during the first 2 years of treatment when prednisone dose is reduced to 5–10 mg/day [6].

Over the last few decades, non-biologic (mainly methotrexate-MTX) and biologic (anti-interleukin-6, IL-6 such as tocilizumab-TCZ) therapies have been used in clinical practice to minimize GC use and their side effects while close monitoring and treatment of various comorbidities in these patients is implemented [8, 9]. The effects of these interventions in the rates of GC discontinuation and disease relapses have not been thoroughly examined.

The aim of our study was to evaluate the rate of GC discontinuation and the predictive factors for relapses in a contemporary cohort of patients with GCA.

## Material and methods

### Patients

This was a multicenter, prospective cohort study of patients with GCA held by the GCA study group of the Greek Rheumatology Society (ERE-EPERE) [10]. Participating centers included academic and non-academic referral rheumatology clinics, National Health System outpatient clinics and private offices. Ethical approval was provided by the local institutional boards of participating centers and informed consent was obtained from participating patients.

Between December 2015 and December 2019, GCA patients were cross-sectionally evaluated (1st evaluation) in each center, outpatient clinic or private office. The diagnosis of GCA was based on the 1990 American College of Rheumatology (ACR) classification criteria [11]. Patients were excluded if they were not routinely followed up by their physician or had less than 2 visits between the 1st and 2nd evaluation. For each patient, data regarding patient (age, gender, weight, height, working status, educational status, smoking, and alcohol habits) and disease (disease duration and presenting symptoms, laboratory, imaging of temporal and large vessels and temporal artery biopsy data) characteristics as well as treatment patterns (GC dose, use of non-biologic and biologic therapies, treatment adverse events) were recorded and analyzed. Data regarding the mode of diagnosis (temporal artery biopsy, ultrasound of the temporal arteries, large vessel imaging) were collected retrospectively. These were performed at each center’s according to the caring physician discretion.

Comorbidities, including hyperlipidemia, coronary artery disease, cerebrovascular disease, peripheral vascular disease, diabetes mellitus, chronic obstructive pulmonary disease (COPD), arterial hypertension, depression, osteoporosis, current or past hepatitis B virus (HBV) infection, current or past hepatitis C virus (HCV) infection, history of tuberculosis (TB) or latent TB infection (LTBI), history of herpes zoster (HZ), neoplastic diseases and history of vaccination against influenza and pneumococcus were also documented. The diagnosis of each comorbidity was made based on the use of prescribed treatment for its management.

Patients were followed by their physicians and the same data were collected approximately 24 months (2 years) later at the 2nd evaluation. The number and type of relapses during this follow-up period were recorded. Relapse was defined as reappearance of disease related symptoms, usually accompanied by elevation of acute-phase reactants or evidence of vasculitis in imaging studies (MRI, PET/CT, CTA) that required treatment adjustment.

### Statistical analysis

All analyses were performed with the use of Microsoft Excel 2013 and IBM SPSS Statistics v.20 software. At firsts, data were analyzed by descriptive statistics. Continuous variables were presented by mean and standard deviation if normally distributed and median and interquartile range if otherwise. Categorical variables were presented by counts and percentages. Multivariable logistic regression analysis was used to determine factors associated with GC discontinuation. Age, sex, disease duration, initial GC dose, treatment with biologic or non-biologic agents, and the occurrence of GC related adverse events, disease relapses and new cardiovascular disease (CVD) events during follow-up were used as independent variables. Multivariable logistic regression analysis was additionally used to determine possible predictors of new relapses. Age, sex, disease duration, treatment status, specific treatment with biologic or non-biologic agents, large vessel vasculitis at diagnosis, erythrocyte sedimentation rate at diagnosis and the occurrence of new CVDs during follow-up were used as independent variables. The selection method in the regression analyses was based on the results of initial univariate regression analyses following the Collett’s model selection approach. Clinically significant variables were finally inserted in the model. The final model included: age, sex, duration of disease, treatment, prednisolone dose, treatment with GC-sparing agents, treatment with biologics, large vessel vasculitis at diagnosis, erythrocyte sedimentation rate at diagnosis, cardiovascular events during follow-up. Collinearity was excluded using the Variance Inflation Factor measure.

## Results

### Patient and disease characteristics at baseline

Initially, 243 GCA patients were included in the study. 67% were women with a mean age at diagnosis of 72 years (Table 1) and a median disease duration of 2 years at the 1st evaluation. Temporal artery biopsy had been performed in 196 patients (81% of the whole cohort) and was positive in 161 of them (82%). 103 patients (43%) underwent temporal artery ultrasound that was positive in half of them (51%). Furthermore, large vessel involvement indicated by positive imaging studies (MRA, PET/CT, CTA) was found in 7% of the patients.

**Table 1:** Patient and disease characteristics

Patient characteristics	*n* = 243
Females, *n* (%)	162 (67)
Age at diagnosis, years, mean ± 1SD	72.1 ± 8.1
Disease duration at 1st evaluation, years, median (IQR)	2(3)
Working status, retired, *n* (%)	201 (83)
BMI, mean ± 1SD	26.4 ± 4.0
Current smokers, *n* (%)	29 (13)
Previous smokers, *n* (%)	58 (27)
Alcohol, *n* (%)	
≥ 2 times/month	135 (62)
0–1 times/month	46 (21)
Method of diagnosis	
Temporal artery biopsy, *n* positive/total (%)	161/196 (82)
Ultrasound of the temporal arteries, *n* positive/total (%)	53/103 (51)
Large vessel imaging, *n* positive/total (%)	16/238 (7)
Symptoms at diagnosis, *n* (%)	
Headache	173 (71)
Fever	157 (65)
Jaw claudication	83 (34)
Scalp tenderness	78 (32)
Polymyalgia rheumatica symptoms	77 (32)
Visual disturbances	50 (21)
Laboratory findings at diagnosis	
ESR, mm/h, median (IQR)	102 (36)
CRP, mg/L, median (IQR)	65.8 (92)
Comorbidities at diagnosis, *n* (%)	
Coronary artery disease	19 (8)
Stroke	9 (4)
Peripheral artery disease	15 (6)
Arterial hypertension	142 (58)
Hyperlipidemia	79 (33)
Diabetes mellitus	44 (18)
Current neoplastic diseases	5 (2)
Osteoporosis	114 (47)
Depression	27 (11)
Chronic obstructive pulmonary disease	18 (7)

*CRP* C-reactive protein, *ESR* erythrocyte sedimentation rate, *BMI* body mass index, *SD* standard Deviation

The most common symptoms at diagnosis are shown in Table 1. Visual disturbances were present in 21% of patients. The median erythrocyte sedimentation rate (ESR) and C-reactive protein (CRP) at diagnosis were 102 mm/h (IQR 36) and 65.8 mg/L (IQR 92) respectively (Table 1). Regarding co-morbidities, a history of coronary artery disease, stroke and peripheral artery disease was reported, based on history and/or therapy, in 8%, 4% and 6% of patients, respectively. Moreover, arterial hypertension was reported in 58%, hyperlipidemia in 33% and diabetes mellitus in 18% of patients while current neoplastic diseases, depression, osteoporosis (defined by anti-osteoporotic therapy and/or history of osteoporotic fractures) and COPD were reported in 2%, 11%, 47% and 7% respectively. Regarding vaccination, 85 (33.5%) patients had anti-pneumococcal vaccination and 158 (63%) had been vaccinated for influenza. Four patients (2%) had active HBV infection (HBsAg positive) at the time of diagnosis, while no patient with HCV infection was reported. Finally, LTBI was identified in 8 patients (Mantoux test positive).

### Treatment patterns

All patients were treated with GCs at diagnosis. Seventeen patients (6.7%) with visual disturbances received intravenous pulses of methylprednisolone (1000 mg/day for 3 days). The initial median prednisolone dose was 40 mg/day. Only 8 patients (3%) were also treated with MTX and none with biologics at diagnosis. At the 2nd evaluation 66 patients were receiving GCs as  monotherapy and 26 patients were receiving GCs in combination with non-biologics/biologics (Table 2).

**Table 2:** Treatment patterns at different time points during the disease course

Treatment	Diagnosis	1st evaluation	2nd evaluation
Glucocorticoids, *n* (%)	243/243 (100%)	194/243 (80%)	92/160 (58%)
Monotherapy	235	142	66
Combination with biologics/non-biologics	8	52	26
Non-biologics, *n* (%)	8/243 (3%)	49/243 (20%)	27/160 (17%)
MTX	8	49	26
Biologics, *n* (%)	0/243 (0%)	18/243 (7.5%)	21/160 (13%)
TCZ	0	18	18
Infliximab	0	0	1
Abatacept	0	0	2
Combination of biologics and non-biologics, *n* (%)	0/243 (0%)	4/243 (1.7%)	2/160 (1%)

*MTX* methotrexate, *TCZ* tocilizumab

**Fig. 1:**
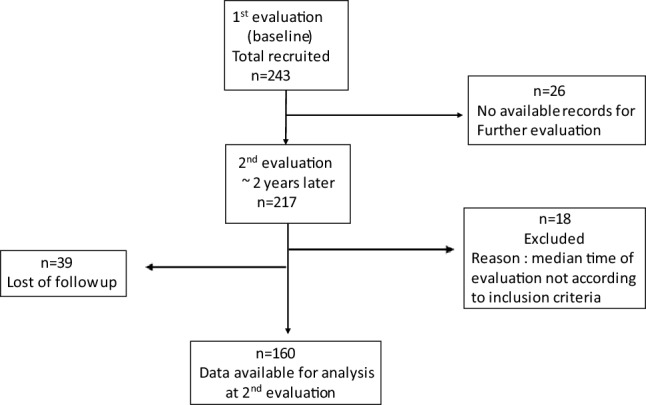
Flowchart of patient disposition

In Fig. 1, the flowchart of patients is shown. At the 2nd evaluation, complete data were available for 160 patients (66%) with a median interval between the 1st and 2nd evaluation of 2.02 years (Fig. 1). Eighteen patients were excluded as the median time of the 2nd evaluation was less (< 1 year) or exceeded (> 3 years) the suggested reevaluation time point (2 years after the 1st evaluation) while 39 patients were lost to follow-up. No differences were observed between patients who were lost to follow-up and those who were analyzed after 2 years regarding patient characteristics (*p* = 0.89 for sex, *p* = 0.92 for age), disease characteristics (*p* = 0.18 for LVV involvement, *p* = 0.18 for ESR and *p* = 0.50 for disease duration) and treatment patterns (*p* = 0.92 for non-biologics, *p* = 0.84 for biologics and *p* = 0.44 for glucocorticoids).

Regarding treatment with non-biologics, 20% (49/243) were receiving MTX at 1st evaluation and a similar percentage 2 years later (16%, 26/160, Table 2) while for biologics 7.5% were receiving them at 1st evaluation and 13% 2 years later, respectively. Among patients who had received biologics, 18 were on TCZ, 2 on abatacept and 1 on infliximab (Table 2).

### Rates and factors associated with GC discontinuation

At the end of the follow-up (median time since diagnosis: 4.3 years), 58% were still on GCs (median dose: 5 mg/day) while 28.7% were on a GC-sparing agent (biologic or non-biologic). No factors were identified by multivariable logistic regression analysis to be associated with GC discontinuation. There was a marginally non-statistically significant negative association between age and GC discontinuation [odds ratio 0.958 (0.916, 1.003) per 1-year increase, *p* = 0.065).

### Rates and predictors of relapses

During the prospective 2-year follow-up period, 43 patients (27%) suffered ≥ 1 relapse (relapse rate: 17/100 patient-years). The median time from the 1st evaluation to the 1st relapse was 8 months. These were mainly clinical relapses (75.5% in 47.2% accompanied by increases in ESR and/or CRP) while in 22.6% the relapse was assumed by laboratory findings while in 1 patient (1.9%) the diagnosis was made by suggestive large vessel imaging findings. Relapses were managed by increasing the dose of GCs (*n* = 33.61%) or adding a GC-sparing agent (MTX, *n* = 4 or TCZ, *n* = 5).

Patients who relapsed (*n* = 43) compared to those who did not relapse (*n* = 117) had shorter disease duration (median 1.4 vs. 3 years at 1st evaluation, *p* = 0.015), more frequent large vessel involvement (21% vs. 8%, *p* = 0.019), had received a higher GC dose at diagnosis (mean daily prednisolone dose: 46.2 vs*.* 39.9 mg, *p* = 0.01) and were more likely to have developed a CVD during the 2-year follow-up period (14% vs. 4.2%, *p* = 0.032, Table 3).

**Table 3:** . Main characteristics of relapsing *vs.* non-relapsing patients

	Relapses		*p* value
	No	Yes	
	*n* = 117	*n* = 43	
Age at diagnosis, years (mean ± SD)	72.3 ± 7.9	72.7 ± 8.2	0.82
Females, *n* (%)	74 (65%)	30 (73%)	0.33
Disease duration at 1st evaluation (years)	**3 (4.38)**	**1.4 (2.5)**	**0.015**
CRP at diagnosis (mg/L)	51 (82)	51.4 (125)	0.42
ESR at diagnosis (mm/h)	105 (33)	102 (48.75)	0.93
Large vessel involvement, *n* (%)	**9 (8%)**	**9 (21%)**	**0.019**
CVD events between 1st and 2nd evaluation, *n* (%)	**5 (4.2%)**	**6 (14%)**	**0.032**
GC starting dose at diagnosis (mean, mg/day)	**39.9 ± 11.8**	**46.2 ± 13.4**	**0.01**
Therapies at 1st evaluation			
Off therapy, *n* (%)	18 (16%)	1 (2%)	0.066
GCs alone, *n* (%)	68 (59%)	28 (65%)	0.34
GC and GC-sparing agents (biologics, non-biologics), *n* (%)	30 (26%)	14 (33%)	0.38
Biologics, *n* (%)	7 (6%)	6 (14%)	0.1

*SD* standard deviation, *CRP* C-reactive protein, *ESR* erythrocyte sedimentation rate, *CVD* cardiovascular disease, *GC* glucocorticoids

Characteristics with a *p* value < 0.05 are shown in bold

By multivariable logistic regression analysis, large vessel involvement [odds ratio (OR) (95% confidence intervals) = 4.22 (1.14, 15.58), *p* = 0.03], a new CVD event [OR = 4.60 (1.11, 19.13), *p* = 0.04] and a higher GC dose at diagnosis [OR = 1.04 (1.002, 1.08), *p* = 0.04] were associated with increased odds for a relapse (Table 4).

**Table 4:** Variables associated with relapses by multivariable logistic regression analysis

Variable	Odds ratio (95% CI)	*p* value
Age	1.04 (0.98, 1.10)	0.20
Female sex	1.73 (0.68, 4.40)	0.25
Duration of the disease	0.85 (0.70, 1.03)	0.09
Treatment (any treatment for GCA)	2.79 (0.29, 26.66)	0.37
Prednisolone, initial dose at diagnosis (mg/day)	**1.04 (1.002, 1.08)**	**0.04**
Treatment with GC-sparing agents (biologics or non-biologics)	1.05 (0.33, 3.30)	0.94
Treatment with biologics	2.30 (0.38, 13.83)	0.37
Large vessel vasculitis at diagnosis	**4.22 (1.14, 15.58)**	**0.03**
ESR at diagnosis (mm/h)	1.00 (0.98, 1.01)	0.64
CVD events during follow-up	**4.60 (1.11, 19.13)**	**0.04**

*CI* confidence intervals, *GCA* giant cell arteritis, *CVD* cardiovascular event, *ESR* erythrocyte sedimentation rate

Characteristics with a *p* value < 0.05 are shown in bold

### Serious events

During the 2-year follow-up period, 43 GC-related adverse events were recorded. The most common was osteoporosis occurring in 20 patients (12.5%) while 11 patients (6.9%) had 12 documented fractures. Other GC-related side effects included cataract (*n* = 9, 5.6%) and diabetes mellitus (*n* = 3, 1.9%). Hospitalizations were mainly due to CVD events (*n* = 11, 6.9% such as myocardial infarction/*n* = 2, rupture of aortic aneurysm/n = 2 and heart failure/ *n* = 2) and serious infections (*n* = 9, 5.6%). Herpes zoster was reported in 3.1% of patients and malignancies in 4.4% (lung cancer, *n* = 2 and non-melanoma skin cancer, *n* = 2).

During follow-up, 10 patients died (6.2%). The exact cause of death was available for eight patients, and it was due to cardiovascular events (*n* = 3, 30%), infections (*n* = 2, 20%) or other causes (*n* = 3, 30%).

## Discussion

This is one of the few real-life studies examining the relapse and drug discontinuation rates in patients with GCA in the contemporary treatment era. Our findings indicate that there was a trend for a lower relapse and a similar GC discontinuation rate compared to older GCA cohorts. Factors associated with relapses included large vessel involvement, initial GC dose and CVD events during follow-up.

GCA has been traditionally treated with GCs while non-biologic (mainly MTX) and biologic (anti-IL6, TCZ) agents are usually employed for refractory or relapsing disease. Recent Guidelines and Recommendations propose their use in combination with GCs at diagnosis either for patients who are at risk for GC-related side effects or complications (EULAR) or for all patients (ACR). In both cases, TCZ is the preferred GC-sparing agent compared to MTX. For patients who had not received TCZ at diagnosis, EULAR recommends them for relapsing or refractory to GC disease. Real-life data though regarding the course of patients (relapses, GC-discontinuation rate) with GCA with the addition of these agents are limited.

In our study, 37% of patients received non-biologic and 16% biologic agents during their disease course. During the entire 4-year follow-up period, 42% of patients were able to discontinue GCs compared to 24–55% in older GCA cohorts [7, 12, 13]. While in older studies a number of factors were associated with a higher GC-discontinuation rate such as a higher initial oral prednisone dose [7], a higher hemoglobin level [14], the use of GC-sparing agents and male sex [15], in our study no baseline or on therapy factors were associated with GC discontinuation. There was only a non-statistically significant trend for younger patients to achieve GC discontinuation.

Long-term GC treatment is associated with the development of several well-recognized adverse events [5, 16, 17]. In our study, 22% developed GC-related adverse events including cataract and osteoporosis over a 2-year period. This rate is rather lower compared to the 46% rate reported by Labarca et al. during the same follow-up period and similar to that observed by Proven et al. (86% during 10 years of follow-up) [7, 18].

It is currently unknown whether the gradual introduction of non-biologic and biologic agents in the treatment of GCA has resulted in a decreased relapse rate. In our study, relapses occurred in 27% of patients during the 2-year prospective follow-up period (17/100 patient-years). Although direct comparisons cannot be made, it seems that this relapse rate is lower compared to what has been reported in previous GCA cohorts [6, 7, 12, 14, 15, 18–22]. Several studies report that the first 2 years after diagnosis 35% of patients relapse [14, 21] while a higher percentage is observed (40–52%) during the 1st year after diagnosis when GCs are tapered [12, 19, 22]. In a cohort of patients with a total follow-up of approximately 5 years after diagnosis, Labarca et al. observed that 79% of patients experienced ≥ 1 relapse [7].

Numerous patient and/or disease characteristics have been associated with GCA relapses. These included clinical characteristics such as jaw claudication, scalp tenderness, polymyalgia rheumatica symptoms [6, 15] or fever ≥ 38 °C [14], the severity of inflammatory infiltrate in temporal artery biopsy [14], female sex [7, 15], specific comorbidities like diabetes mellitus, hypertension and anemia [7, 19] and a strong initial inflammatory response (defined by the presence of fever, weight loss, ESR > 85 mm/h and hemoglobin < 110 gm/L) [23, 24]. It is obvious from these studies that a consistent phenotype associated with relapses has not been defined.

In our study, large vessel involvement was associated with an almost a fourfold higher risk for disease relapses. This is in accordance with the findings from other recent studies [21, 22, 25, 26], indicating that it should be explored further whether all patients with GCA should be screened for large vessel involvement at diagnosis and receive a more aggressive treatment scheme to prevent relapses and decrease the rate of thoracic aneurysm formation, as is the case with another large vessel arteritis (Takayasu arteritis) [8, 9].

A novel finding of our study was that patients who had a new CVD event during follow-up had a higher relapse risk. In general, patients with GCA are at higher risk for CVD events due to their advanced age and cardiovascular risk factors at diagnosis as well as to disease-related factors [22, 27, 28]. In our cohort, the mean age of patients at diagnosis was 72 years while a substantial number had traditional CVD risk factors (58.3% hypertension, 33% hyperlipidemia, 18% diabetes mellitus). It is unclear if this higher relapse rate could be related to a less aggressive use of GCs in patients with new CVD events during follow-up or to other unknown factors. Nevertheless, this is a finding that needs to be replicated in larger patient cohorts.

Another interesting finding was that there was a dose-dependent increase in the relapse rate according to the initial GC dose (OR = 1.04). Previous studies by Restuccia et al. [14] and Martinez-Lado et al. [19] as well as a recent metanalysis [20] have not shown such an association. A possible explanation could be that patients with more severe presentation (i.e., vision involvement, higher inflammatory response) may require higher initial GC doses and are more prone to relapses. This hypothesis is supported by the findings from a study of Hernandez-Rodriguez et al. in which patients with a strong inflammatory response (fever, weight loss, ESR > 85 mm/h and hemoglobulin < 110 gm/L) had higher GC requirements but also more disease flares [23].

### Limitations and strengths

Major strengths of our study include its real-life multi-center design, the large number of included patients, the utilization of invasive and non-invasive modes of GCA diagnosis, its 2-year prospective follow-up period and the inclusion of several referral centers with significant experience in patients with GCA. On the other hand, our study has certain limitations. As is the case with similar multi-center, real-life patient cohorts, a significant number of patients (34% from the initial cohort) were lost in follow-up while there was no universally employed protocol for the diagnosis, monitoring, and management of these patients among different centers.

## Conclusion

Our contemporary, real-life cohort study demonstrates that after the gradual introduction of GC-sparing agents in GCA management, there was a trend for a lower relapse rate compared to older cohorts. Large vessel involvement and new CVD events were associated with relapses indicating the need for replicating these findings in larger patient cohorts that could lead to a different diagnostic and treatment approach in this patient population. Furthermore, we noted that the GC discontinuation rate has not been altered significantly, since ~ 60% of patients remained on them 4 years after diagnosis. These findings emphasize the need for more real-life studies evaluating the earlier use of non-GC therapies as well as for the testing and introduction of novel GC-sparing agents in GCA treatment.

## Data Availability

Data are available from the corresponding author upon reasonable request.

## References

[1] Salvarani C, Pipitone N, Versari A, Hunder GG (2012). Clinical features of polymyalgia rheumatica and giant cell arteritis. Nat Rev Rheumatol.

[2] Dejaco C, Duftner C, Buttgereit F, Matteson EL, Dasgupta B (2017). The spectrum of giant cell arteritis and polymyalgia rheumatica: revisiting the concept of the disease. Rheumatology (Oxford).

[3] Salvarani C, Cimino L, Macchioni P, Consonni D, Cantini F, Bajocchi G, Pipitone N, Catanoso MG, Boiardi L (2005). Risk factors for visual loss in an Italian population-based cohort of patients with giant cell arteritis. Arthritis Rheum.

[4] Buttgereit F, Dejaco C, Matteson EL, Dasgupta B (2016). Polymyalgia rheumatica and giant cell arteritis: a systematic review. JAMA.

[5] Matteson EL, Buttgereit F, Dejaco C, Dasgupta B (2016). Glucocorticoids for management of polymyalgia rheumatica and giant cell arteritis. Rheum Dis Clin N Am.

[6] Alba MA, Garcia-Martinez A, Prieto-Gonzalez S, Tavera-Bahillo I, Corbera-Bellalta M, Planas-Rigol E, Espigol-Frigole G, Butjosa M, Hernandez-Rodriguez J, Cid MC (2014). Relapses in patients with giant cell arteritis: prevalence, characteristics, and associated clinical findings in a longitudinally followed cohort of 106 patients. Medicine (Baltimore).

[7] Labarca C, Koster MJ, Crowson CS, Makol A, Ytterberg SR, Matteson EL, Warrington KJ (2016). Predictors of relapse and treatment outcomes in biopsy-proven giant cell arteritis: a retrospective cohort study. Rheumatology (Oxford).

[8] Maz M, Chung SA, Abril A, Langford CA, Gorelik M, Guyatt G, Archer AM, Conn DL, Full KA, Grayson PC, Ibarra MF, Imundo LF, Kim S, Merkel PA, Rhee RL, Seo P, Stone JH, Sule S, Sundel RP, Vitobaldi OI, Warner A, Byram K, Dua AB, Husainat N, James KE, Kalot MA, Lin YC, Springer JM, Turgunbaev M, Villa-Forte A, Turner AS, Mustafa RA (2021). 2021 American College of Rheumatology/Vasculitis Foundation Guideline for the management of giant cell arteritis and takayasu arteritis. Arthritis Care Res (Hoboken).

[9] Hellmich B, Agueda A, Monti S, Buttgereit F, de Boysson H, Brouwer E, Cassie R, Cid MC, Dasgupta B, Dejaco C, Hatemi G, Hollinger N, Mahr A, Mollan SP, Mukhtyar C, Ponte C, Salvarani C, Sivakumar R, Tian X, Tomasson G, Turesson C, Schmidt W, Villiger PM, Watts R, Young C, Luqmani RA (2020). 2018 Update of the EULAR recommendations for the management of large vessel vasculitis. Ann Rheum Dis.

[10] Tsalapaki C, Nikitopoulou E, Boki KA, Boumpas D, Sfikakis PP, Vosvotekas G, Voulgari PV, Vassilopoulos D (2018). Five-year prospective multi-center cohort study of patients with giant cell arteritis in Greece. Mediterr J Rheumatol.

[11] Hunder GG, Bloch DA, Michel BA, Stevens MB, Arend WP, Calabrese LH, Edworthy SM, Fauci AS, Leavitt RY, Lie JT (1990). The American College of Rheumatology 1990 criteria for the classification of giant cell arteritis. Arthritis Rheum.

[12] Perrineau S, Ghesquière T, Charles P, Paule R, Samson M, Gayraud M, Chauvin A, Terrier B, Guillevin L, Bonnotte B, Mouthon L, Régent A (2021). A French cohort of patients with giant cell arteritis: glucocorticoid treatment and its associated side effects. Clin Exp Rheumatol.

[13] Albrecht K, Huscher D, Buttgereit F, Aringer M, Hoese G, Ochs W, Thiele K, Zink A (2018). Long-term glucocorticoid treatment in patients with polymyalgia rheumatica, giant cell arteritis, or both diseases: results from a national rheumatology database. Rheumatol Int.

[14] Restuccia G, Boiardi L, Cavazza A, Catanoso M, Macchioni P, Muratore F, Cimino L, Aldigeri R, Crescentini F, Pipitone N, Salvarani C (2016). Flares in biopsy-proven giant cell arteritis in Northern Italy: characteristics and predictors in a long-term follow-up study. Medicine (Baltimore).

[15] Unizony SH, Bao M, Han J, Luder Y, Pavlov A, Stone JH (2021). Treatment failure in giant cell arteritis. Ann Rheum Dis.

[16] Wilson JC, Sarsour K, Collinson N, Tuckwell K, Musselman D, Klearman M, Napalkov P, Jick SS, Stone JH, Meier CR (2017). Serious adverse effects associated with glucocorticoid therapy in patients with giant cell arteritis (GCA): a nested case-control analysis. Semin Arthritis Rheum.

[17] Petri H, Nevitt A, Sarsour K, Napalkov P, Collinson N (2015). Incidence of giant cell arteritis and characteristics of patients: data-driven analysis of comorbidities. Arthritis Care Res (Hoboken).

[18] Proven A, Gabriel SE, Orces C, O'Fallon WM, Hunder GG (2003). Glucocorticoid therapy in giant cell arteritis: duration and adverse outcomes. Arthritis Rheum.

[19] Martinez-Lado L, Calvino-Diaz C, Pineiro A, Dierssen T, Vazquez-Rodriguez TR, Miranda-Filloy JA, Lopez-Diaz MJ, Blanco R, Llorca J, Gonzalez-Gay MA (2011). Relapses and recurrences in giant cell arteritis: a population-based study of patients with biopsy-proven disease from northwestern Spain. Medicine (Baltimore).

[20] Mainbourg S, Addario A, Samson M, Puechal X, Francois M, Durupt S, Gueyffier F, Cucherat M, Durieu I, Reynaud Q, Lega JC (2020). Prevalence of giant cell arteritis relapse in patients treated with glucocorticoids: a meta-analysis. Arthritis Care Res (Hoboken).

[21] Kermani TA, Warrington KJ, Cuthbertson D, Carette S, Hoffman GS, Khalidi NA, Koening CL, Langford CA, Maksimowicz-McKinnon K, McAlear CA, Monach PA, Seo P, Merkel PA, Ytterberg SR (2015). Disease relapses among patients with giant cell arteritis: a prospective longitudinal cohort study. J Rheumatol.

[22] Dumont A, Parienti JJ, Delmas C, Boutemy J, Maigne G, Martin Silva N, Sultan A, Planchard G, Aouba A, de Boysson H (2020). Factors associated with relapse and dependence on glucocorticoids in giant cell arteritis. J Rheumatol.

[23] Hernández-Rodríguez J, García-Martínez A, Casademont J, Filella X, Esteban MJ, López-Soto A, Fernández-Solà J, Urbano-Márquez A, Grau JM, Cid MC (2002). A strong initial systemic inflammatory response is associated with higher corticosteroid requirements and longer duration of therapy in patients with giant-cell arteritis. Arthritis Rheum.

[24] García-Martínez A, Hernández-Rodríguez J, Espígol-Frigolé G, Prieto-González S, Butjosa M, Segarra M, Lozano E, Cid MC (2010). Clinical relevance of persistently elevated circulating cytokines (tumor necrosis factor alpha and interleukin-6) in the long-term followup of patients with giant cell arteritis. Arthritis Care Res (Hoboken).

[25] Sugihara T, Hasegawa H, Uchida HA, Yoshifuji H, Watanabe Y, Amiya E, Maejima Y, Konishi M, Murakawa Y, Ogawa N, Furuta S, Katsumata Y, Komagata Y, Naniwa T, Okazaki T, Tanaka Y, Takeuchi T, Nakaoka Y, Arimura Y, Harigai M, Isobe M (2020). Associated factors of poor treatment outcomes in patients with giant cell arteritis: clinical implication of large vessel lesions. Arthritis Res Ther.

[26] Espitia O, Néel A, Leux C, Connault J, Espitia-Thibault A, Ponge T, Dupas B, Barrier JH, Hamidou MA, Agard C (2012). Giant cell arteritis with or without aortitis at diagnosis. A retrospective study of 22 patients with longterm followup. J Rheumatol.

[27] Ray JG, Mamdani MM, Geerts WH (2005). Giant cell arteritis and cardiovascular disease in older adults. Heart.

[28] Arias M, Heydari-Kamjani M, Kesselman MM (2021). Giant cell arteritis and cardiac comorbidity. Cureus.

